# Leishmaniasis in Ancient Egypt and Upper Nubia

**DOI:** 10.3201/eid1210.060169

**Published:** 2006-10

**Authors:** Albert R. Zink, Mark Spigelman, Bettina Schraut, Charles L. Greenblatt, Andreas G. Nerlich, Helen D. Donoghue

**Affiliations:** * Academic Teaching Hospital München-Bogenhausen, Munich, Germany;; † Ludwig-Maximilians-Universität München, Munich, Germany;; ‡ University College London, London, UK;; § The Hebrew University, Hadassah Medical School, Jerusalem, Israel

**Keywords:** leishmaniasis, ancient Egypt, Upper Nubia, letter, mummies, paleopathology, letter

**To the Editor:** Leishmaniasis is a disease caused by parasites of the genus *Leishmania*. The infection is transmitted to humans through the bites of female sandflies and manifests mainly in 3 forms: visceral, cutaneous, and mucocutaneous. Visceral leishmaniasis or kala-azar, the often fatal form of the disease, is caused by species of the *Leishmania donovani* complex. These parasites were responsible for severe recent outbreaks in Sudan and other countries and are thought to originate in East Africa (*1**–**4*).

In this report, we describe the successful amplification of *L. donovani* DNA in ancient Egyptian and Christian Nubian mummies dating back 4,000 years. Besides the first proof for visceral leishmaniasis in paleopathology, we provide evidence that leishmaniasis was present in Nubia in the early Christian period and that the organism also infected ancient Egyptians, probably because of close trading contacts to Nubia, during the Middle Kingdom. We analyzed 91 bone tissue samples from ancient Egyptian mummies and skeletons and 70 bone marrow samples from naturally mummified human remains from Upper Nubia. The Egyptian material derived from the Pre- to Early Dynastic site of Abydos (n = 7; 3500–2800 BC), a Middle Kingdom tomb in Thebes West (42; 2050–1650 BC), and different tomb complexes in Thebes West, which were built and used between the Middle and New Kingdom until the Late Period (42; c. 2050–500 BC). The Nubian samples were taken before the flooding caused by the Aswan Dam from 2 early Christian burial sites at Kulubnarti, between the second and third cataracts of the Nile River in northern Sudan. One site was on an island in the Nile and dated from 550 to 750 AD. The other was on the western bank of the Nile and was in use from c.750 to 1500 AD. All samples were tested for *Leishmania* spp. DNA and further characterized by direct sequencing.

In 4 of the 91 Egyptian and 9 of the 70 Nubian samples, a 120-bp fragment of a conserved region of the minicircle molecule of kinetoplastid mitochondrial DNA of the parasite (*5**,**6*) could be successfully amplified and, with the first primer pair, unambiguously related to *L. donovani* species after sequencing (Figure). The positive samples from ancient Egypt exclusively originated from the Middle Kingdom tomb, while no molecular evidence for ancient *Leishmania* DNA was found in the Pre- to Early Dynastic and the New Kingdom to Late Period specimens.

**Figure:**
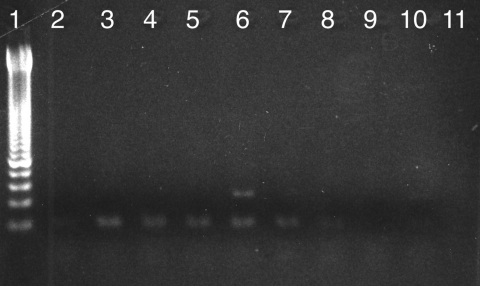
PCR amplification of a 120-bp fragment of kinetoplastid mitochondrial DNA of *Leishmania* spp. in Egyptian and Nubian mummies. Lane 1, 50-bp ladder lanes 2–8, mummy samples; lanes 9,10, extraction controls; lane 11, PCR controls. Lane 6 provides a positive amplification product of the expected size.

In the Middle Kingdom, the Egyptians extended trade relationships and military expeditions to Nubia, the modern Sudan, with particular interest in the gold resources of the country and in obtaining slaves to serve as servants or soldiers in the pharaoh’s army. Today, the Sudan is one of the highly endemic countries for visceral leishmaniasis or kala-azar, which is thought to have originated in East Africa and later spread to the Indian subcontinent and the New World (*4*). Therefore, the high incidence of *Leishmania* DNA in the Middle Kingdom samples (4 [9.5%] of 42) and the lack of findings in earlier or later time periods, may indicate that leishmaniasis was introduced into Egypt at this time. Leishmaniasis did not likely become endemic in the Egyptian Nile Valley because the disease is closely linked to its vector, the phlebotomine sandfly, and the distribution of Acacia-Balanites woodland (*7*). That ancient Egyptians became infected because of close trade contacts and associated travel with Nubia during the Middle Kingdom seems more plausible. The high frequency of *Leishmania* DNA–positive samples in the Nubian mummies (12.9%) suggests that leishmaniasis was endemic in Nubia during the Early Christian period and, in light of the data on the ancient Egyptian mummies, probably already several thousand years before. Taken together, our results support the theory that Sudan could have been indeed the original focus of visceral leishmaniasis (*4*).

Our study shows a completely new aspect of molecular paleopathology. The detection of ancient pathogen DNA is not only used to identify a certain disease and gain information on its frequency and evolutionary origin but also to trace back cultural contacts and their role in the transmission and spread of infectious diseases.
